# Spatiotemporal Correlation of Epileptiform Activity and Gene Expression *in vitro*

**DOI:** 10.3389/fnmol.2021.643763

**Published:** 2021-03-30

**Authors:** Sophie Schlabitz, Laura Monni, Alienor Ragot, Matthias Dipper-Wawra, Julia Onken, Martin Holtkamp, Pawel Fidzinski

**Affiliations:** ^1^Charité – Universitätsmedizin Berlin, Corporate Member of Freie Universität Berlin, Humboldt-Universität zu Berlin, and Berlin Institute of Health, Department of Neurology with Experimental Neurology, Clinical and Experimental Epileptology, Berlin, Germany; ^2^Charité – Universitätsmedizin Berlin, Corporate Member of Freie Universität Berlin, Humboldt-Universität zu Berlin, and Berlin Institute of Health, Department of Neurosurgery, Berlin, Germany; ^3^Epilepsy-Center Berlin-Brandenburg, Institute for Diagnostics of Epilepsy, Berlin, Germany; ^4^Charité – Universitätsmedizin Berlin, Corporate Member of Freie Universität Berlin, Humboldt-Universität zu Berlin, and Berlin Institute of Health, NeuroCure Cluster of Excellence, Neuroscience Research Center, Berlin, Germany

**Keywords:** epileptiform activity *in vitro*, 4-aminopyridine, intrinsic optical signals, gene expression, *c-Fos*, *Icer*, *mTor*, cell death

## Abstract

Epileptiform activity alters gene expression in the central nervous system, a phenomenon that has been studied extensively in animal models. Here, we asked whether also *in vitro* models of seizures are in principle suitable to investigate changes in gene expression due to epileptiform activity and tested this hypothesis mainly in rodent and additionally in some human brain slices. We focused on three genes relevant for seizures and epilepsy: FOS proto-oncogene (*c-Fos*), inducible cAMP early repressor (*Icer*) and mammalian target of rapamycin (*mTor*). Seizure-like events (SLEs) were induced by 4-aminopyridine (4-AP) in rat entorhinal-hippocampal slices and by 4-AP/8 mM potassium in human temporal lobe slices obtained from surgical treatment of epilepsy. SLEs were monitored simultaneously by extracellular field potentials and intrinsic optical signals (IOS) for 1–4 h, mRNA expression was quantified by real time PCR. In rat slices, both duration of SLE exposure and SLE onset region were associated with increased expression of *c-Fos* and *Icer* while no such association was shown for *mTor* expression. Similar to rat slices, *c-FOS* induction in human tissue was increased in slices with epileptiform activity. Our results indicate that irrespective of limitations imposed by *ex vivo* conditions, *in vitro* models represent a suitable tool to investigate gene expression. Our finding is of relevance for the investigation of human tissue that can only be performed *ex vivo*. Specifically, it presents an important prerequisite for future studies on transcriptome-wide and cell-specific changes in human tissue with the goal to reveal novel candidates involved in the pathophysiology of epilepsy and possibly other CNS pathologies.

## Introduction

According to the World Health Organization, diseases of the central nervous system represent a major health risk for the world population (World Health Organization, 2006). To address this risk appropriately and to pursue new therapeutic options, it is critical to understand diverse and often complex molecular mechanisms leading to pathology. For decades, basic research in neurobiology together with disease-specific animal models served as a framework to understand fundamental mechanisms of neurological disorders. This effort led to many useful insights, however, the translational and predictive value of animal models has remained limited (Löscher, 2017). In recent years, this gap between animal models and clinical application has received increasing attention. Consequently, more effort has been put into models that reflect the clinical setting more closely and into novel strategies to increase reproducibility in basic research, aiming for improvement of translation (Bauer et al., 2017).

One of the possibilities to bridge differences between animal models and clinical neurology is to use human tissue-derived *ex vivo* models and to investigate functional disease mechanisms in a species-specific manner. So far, the approaches include the use of resected specimen from brain surgery procedures, investigation of post mortem tissue (Verwer et al., 2002) and the recently evolving field of brain organoids (Di Lullo and Kriegstein, 2017). With the exception of brain organoids, functional investigation of human-derived CNS tissue is constrained by the naturally limited life span of *ex vivo* samples. The recent development of organotypic cultures is a promising advance to overcome this conundrum, however, as of now it still poses challenges associated with low reproducibility and large protocol variety (Jones et al., 2016). According to a recent study on disease burden (GBD 2015 Neurological Disorders Collaborator Group, 2017), epilepsy constitutes one of the most common neurological diseases with a prevalence between 0.5 and 1.0% in the developed world (Fiest et al., 2017). Up to one third of individuals afflicted with epilepsy suffer from pharmacoresistance (Chen et al., 2018). Although numerous *in vivo* and *in vitro* animal models established throughout the years increased our knowledge about disease mechanisms, many questions including how to address pharmacoresistance remain unanswered (Becker, 2018). Recent technological advances in the field of transcriptomics and single cell analysis (Becker et al., 2002; Guelfi et al., 2019) made it possible to discover novel disease-specific genetic alterations in epilepsy. Importantly, these findings include not only monogenetic mutations as singular causative factors. Also, seizure-related modulation of gene expression and gene editing as well as epigenetic modulation during epileptogenesis have been reported (Kobow and Blümcke, 2018).

Modulation of gene expression is mostly studied *in vivo*. However, for obvious reasons such approach is not possible in human tissue – in this case only *ex vivo* studies on resected tissue would be possible. To our knowledge, it is not known whether or to what degree *in vitro* models can recapitulate changes in gene expression observed under *in vivo* conditions. Here, in a proof-of-principle study, we aimed to assess the general, species-independent suitability of *in vitro* models of acute seizures to investigate changes in gene expression due to epileptiform activity. In a first step in that direction, we aimed to test our hypothesis mainly in rat tissue, for which modulation of gene expression due to epileptiform activity has been studied *in viv*o before. In addition, we extended our studies to some human slices. We focused on three genes relevant for seizures and epilepsy: FOS proto-oncogene (*c-Fos*), inducible cAMP early repressor (*Icer*), and mammalian target of rapamycin (*mTor*). Expression of these genes and/or the involved pathways has been described before to be positively modulated by seizure activity and, in case of *Icer* and *mTor*, also to be involved in epileptogenesis (Lund et al., 2008; Porter et al., 2008; Szyndler et al., 2009; Zeng et al., 2009; Huang et al., 2010; Barros et al., 2015).

## Materials and Methods

### Rat Slice Preparation

**FIGURE 1:**
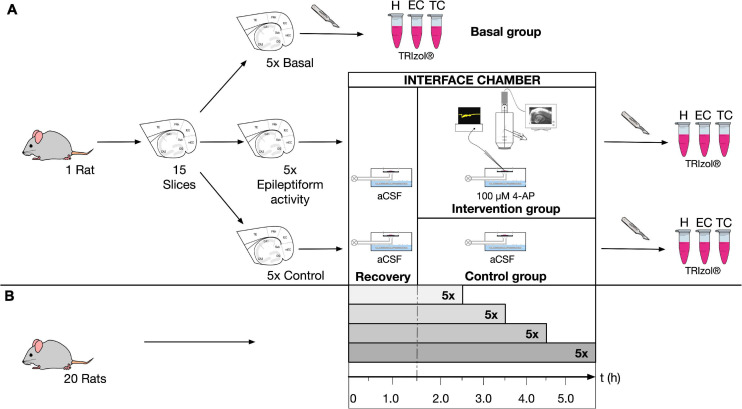
Study design for rat tissue. **(A)** Chronological sequence of each experiment: rat brain preparation; allocation of 15 horizontal slices to three different groups (basal, control and intervention); recovery of 10 slices (control and intervention group) for 1.5 h in an interface chamber with incubation in carbogenated aCSF (95% O_2_, 5% CO_2_, pH 7.4); induction of epileptiform activity by 100 μM 4-AP in five slices (intervention group) and recording of field potentials as well as intrinsic optical signals; five control slices in aCSF for same amount of time; microdissection of slices into hippocampus (H), entorhinal cortex (EC), and temporal cortex (TC) and subsequent cell lysis of pooled tissue in TRIzol before RT-qPCR. **(B)** Procedure was performed with 20 rats in total; five rats were assigned to each 4-AP treatment duration of 1, 2, 3, or 4 h.

All animal procedures were conducted according to the German Animal Welfare Act as well as the European Directive 2010/63/EU for animal experiments and were approved by the Institutional Animal Welfare Officer and the responsible local authority (Landesamt für Gesundheit und Soziales, Berlin, Germany, T0336/12). Institutional security procedures were followed. Experiments were performed using combined hippocampal-entorhinal cortex (EC) slices from rats as previously reported (Heuzeroth et al., 2019) with some modifications. The study design is represented in Figure 1. Briefly, adult male Wistar-Han rats (220–240 g) were deeply anesthetized by inhalation of isoflurane (4% in 100% O_2_) and then decapitated. Their brains were rapidly removed and placed into ice-cold N-methyl-D-glucamine (NMDG) containing artificial cerebrospinal fluid (NMDG-aCSF) (Ting et al., 2014). Equimolar replacement of sodium by NMDG leads to decreased permeation of ions via neuronal membranes and subsequent reduced cell swelling (Hille, 1971). Carbogenated NMDG-aCSF (95% O_2_, 5% CO_2_) contained (in mM): NMDG (93), KCl (2.5), NaH_2_PO_4_ (1.2), NaHCO_3_ (30), MgSO_4_ (10), CaCl_2_ (0.5), glucose (25), HEPES (20), sodium l-ascorbate (5), thiourea (2), and sodium pyruvate (3). Horizontal slices (400 μm) enclosing the hippocampal formation (H), the entorhinal cortex (EC), and adjacent parts of the temporal cortex (TC) were cut using a vibratome (Vibroslicer VT1200S, Leica, Wetzlar, Germany). From each rat brain, 15 slices were collected and assigned in an alternating manner to three groups, five slices each: (1) basal group to determine initial gene expression; (2) control group to investigate effects of slicing and storage; and (3) intervention group in which epileptiform activity was induced. Slices of the control and intervention group were individually placed in an interface chamber and continuously perfused (1.5–2.0 ml/min) with prewarmed (35°C) and carbogenated aCSF (95% O_2_, 5% CO_2_, pH 7.4), containing (in mM): NaCl (129), KCl (3), NaH_2_PO_4_ (1.25), NaHCO_3_ (21), MgSO_4_ (1.8), CaCl_2_ (1.6), and glucose (10). During storage and experiments, warmed, humified carbogen was directed over the slice surface. All slices were allowed to recover 1.5 h after preparation. In the intervention group, seizure-like events (SLEs) were induced by 100 μM 4-aminopyridine (4-AP, Sigma, Munich, Germany), which non-selectively blocks voltage-dependent potassium channels, augments presynaptic calcium influx (Mathie et al., 1998) and enhances synaptic transmission (Perreault and Avoli, 1991). *In vitro* addition of 4-AP to aCSF results in long lasting ictal-like discharges considered as correspondent to focal to bilateral seizures *in vivo* (Avoli et al., 2002). The intervention group was treated with 4-AP for either 1, 2, 3, or 4 h, while the control slices remained in aCSF for the same amount of time. This procedure was performed with 20 rats in total and five rats were assigned to each treatment duration.

### Human Slice Preparation

Brain temporal lobe tissue was obtained from surgical treatment of epilepsy in five female donor patients (age at resection: 21–58 years) diagnosed with mesial temporal lobe epilepsy (mTLE). The experimental protocol was approved by the local Ethics Committee (EA2/111/14) in agreement with the Declaration of Helsinki. Written informed consent was given by all patients before surgery. Cortex specimens were collected in the operating room, immediately immersed in cold (∼4°C) carbogenated NMDG-aCSF (95% O_2_, 5% CO_2_). The same solution was used for transportation and tissue processing, which took in total up to 2 h. 400 μm-thick slices were then sectioned and assigned as basal, control and intervention as described above. Before start of experiments, slices recovered in standard aCSF for 5 h. After the stabilization period, on average three slices per patient were further incubated for the duration of 4 h in standard aCSF (control) or with aCSF containing 8 mM potassium and 100 μM 4-AP (pH 7.4) (intervention) to examine the effects of slicing and storage and epileptiform activity on gene expression, respectively. In human brain slices, the application of neither 4-AP alone nor 10 or 12 mM potassium is sufficient to induce epileptiform activity (Gabriel et al., 2004). Therefore, SLEs were evoked by applying a combination of high potassium (8 mM) and 4-AP (100 μM), while adjusting the increased osmolarity of aCSF by lowering the concentration of NaCl from 129 to 124 mM (Kraus et al., 2019).

### Electrophysiological and Optical Recordings

Epileptiform activity was monitored by local field potential recordings in 20 selected slices of the intervention group (one slice per rat and three slices per human sample). To control for the influence of the slice position, rat slices were classified to different locations along the dorsoventral axis defined as ventral (−7.6 to −6.8 mm ventrally from bregma), medial (−6.4 to −5.6 mm), and dorsal (−5.2 to −4.4 mm) (see also Paxinos and Watson, 1998). Extracellular field potentials were measured with glass electrodes (filled with 150 mM NaCl, electrode resistance 1–2 MΩ). In rats, one electrode was placed in layer IV or V of the lateral EC, the second electrode was positioned in the stratum pyramidale of the hippocampal CA1 region. In human tissue, SLEs were recorded for at least 40 min with one electrode placed on the superficial cortical layers of human slices (layers II/III, 200–700 μm from pial surface) (Köhling and Avoli, 2006). Electrophysiological signals were acquired with a custom-made amplifier (10×) connected to an A/D interface (Micro 1401 mk II, Cambridge Electronic Design Limited, Cambridge, United Kingdom). Data were recorded with Spike2 and Signal (versions 7.00 and 3.07, respectively, Cambridge Electronic Design Limited, Cambridge, United Kingdom) and analyzed using custom-written algorithms in MATLAB (R2014b, MathWorks, Natick, MA, United States). SLEs were identified using the following criteria: (1) field potential decrease > 0.3 mV, (2) duration of field potential shift > 10 s and (3) superimposition by ripple-like discharges during negative field potential shift.

Intrinsic optical imaging was employed in each electrophysiologically recorded slice according to previous reports (Weissinger et al., 2017). In brief, slices were positioned on a transparent membrane (0.4 μm Millicell culture plate inserts, Millipore, Bedford MA, United States) and homogeneously illuminated from below by a halogen cold light source (KL 1500, Schott, Wiesbaden, Germany) and a curved glass rod (Ø 8 mm). Images were received using an upright binocular microscope (MS 5, Leica, Bensheim, Germany) with a 4× objective, a monocular phototube (Leica, Bensheim, Germany) and a CCD camera (8 bit, Sanyo, Osaka, Japan). In-house macros for ImageJ 1.51m9 (Wayne Rasband National Institutes of Health, United States) and MATLAB software were applied for the processing of the images. 8-bit video signals were converted at a 10 MHz ratio into 320 × 240-pixel images employing a frame-grabber board (pciGrabber-4plus, Phytec, Mainz, Germany). Images were only saved when triggered by the experimenter in case ictal activity became apparent in the electrophysiological recording. Using a circular data buffer, the first image was captured 5–10 s before the onset of the SLE, the recording continued for 50–180 s depending on the duration of the electrophysiologically recorded ictal event. The time course of light transmittance was calculated for each SLE as difference in light transmittance (Δ*T*) between a given image and the control image (mean of the first 20 images in each series recorded before start of ictal activity), and expressed as percentage of the control image transmittance (Δ*T*/*T*). During ictal events, Δ*T*/*T* typically ranged from 1.0 to 7.5% whereas background noise never exceeded 1.0%. The amplitude during SLEs is expressed as max(Δ*T*/*T*). Optical imaging also enabled us to detect spreading depolarizations (SDs) associated with decreased light transmittance (Müller and Somjen, 1999). In regions displaying SD, ΔT/T decreased in the range of −1.0 to −9.2%. Changes of light transmittance over time provided insight into onset, direction and evolution of both ictal activity and SDs in the entire slice. Using squared regions of interests (ROI) sized 20 × 20 pixels each, the optical signal was quantified separately for the anatomical regions (H, EC, and TC) assigned to later gene expression analysis. Optical signals were considered significantly associated with a SLE when the increase of light transmittance was above a threshold of >1.0% and with a SD when the decrease of light transmittance was <−1.0%. The onset of SLE activity was determined by the first region where transmittance increase above threshold was observed. For calculation of the SLE area, every pixel within a given anatomical region that reached at least 1% ΔT/T in > 9 subsequent images during one SLE was considered to be involved in this SLE. The SLE area was calculated for each region separately and expressed as percentage of the total area for a given anatomical region.

### RNA Extraction and Reverse Transcription

All investigated rat slices were microdissected into H, EC and TC in order to separately quantify mRNA levels within these brain areas. In human cortical slices, microdissection was aimed to separate the seizure onset region (named “onset site” group) determined by electrophysiology and optical imaging from the rest of the slice (named “rest” group). Basal slices were snap frozen in liquid nitrogen and stored at −80°C immediately after preparation while slices from the intervention and control groups were frozen immediately after the assigned experimental time period (1–4 h in rat and 4 h in human slices). To obtain sufficient amounts of mRNA, tissue from different slices within one experiment but the same anatomical region and group was pooled. Pooled tissue was lyzed in 1 ml TRIzol (Invitrogen AG, Carlsbad, United States). Tissue homogenization and cell lysis were facilitated by highspeed shaking (50 Hz) with stainless steel beads for 10 min (TissueLyser LT, Qiagen N.V., Venlo, Netherlands). Based on the acid guanudinium thiocyanate-phenol-chloroform extraction method, chloroform (200 μl) was added to the sample to isolate RNA (Chomczynski and Sacchi, 1987). Phase separation was conducted by centrifugation (15 min at 12,000 × *g* and 4°C) and subsequent precipitation of RNA by incubation with 500 μl isopropanol for 10 min and 3.5 μl Recombinant RNasin Ribonuclease Inhibitor (Promega, Fitchburg, WI, United States) resuspended RNA in 25.0 μl nuclease-free water. Additional RNA purification was accomplished by refilling the remaining volume to 200 μl and adding an equal volume of Roti-Phenol/Chloroform/Isoamyl alcohol (Carl Roth, Karlsruhe, Germany). Following short-time centrifugation (5 min), the RNA containing aqueous phase was treated with 200 μl chloroform to remove residual phenol from the solution. RNA was then recovered by overnight precipitation with 550 μl ethanol (96%) and 6 μl ammonium acetate (10 M) at −20°C and dissolved in 16 μl nuclease-free water. Concentration of total RNA was determined by measuring the optical density at 260 nm with NanoDrop 2000 Spectrophotometer (Thermo Fisher Scientific Inc., Waltham, MA, United States) and ranged from 143.5 to 738.5 ng/μl per rat sample and from 71.4 to 2,224.2 ng/μl per human sample. Purity was checked using the 260/230 nm as well as the 260/280 nm ratios (accepted values > 1.8). First strand cDNA synthesis was carried out using moloney murine leukemia virus reverse transcriptase (M-MLV RT, Promega, Fitchburg, WI, United States) according to the manufacturer’s instructions with several modifications. In brief, a mixture of 15.0 μl diluted, purified RNA (2.0 μg for rat sample, 0.5 μg for human sample), 1.5 μl random hexamers (100 μM, Roche, Basel, Switzerland) and 1.5 μl dNTPs (10 mM each, Roche, Basel, Switzerland) was incubated at 65°C for 5 min. The mixture was then assembled with a prepared reaction volume, consisting of: 2.0 μl M-MLV-RT (200 U/μl), 5.0 μl M-MLV RT Reaction Buffer (Promega, Fitchburg, WI, United States), 0.5 μl Recombinant RNasin Ribonuclease Inhibitor (40 U/μl) and 0.5 μl DTT (100 mM, Promega, Fitchburg, WI, United States). Reverse transcription mixture was successively incubated as follows: 5 min at 21°C, 60 min at 37°C, 15 min at 70°C. To assess genomic DNA contamination in the RNA preparation, a minus RT-control was included in each quantitative reverse transcription PCR (RT-qPCR) experiment. cDNA samples were stored at −20°C until further analysis.

### Primer Design and Efficiency

Quantitative PCR (qPCR) primer design was based on mRNA sequences from the RefSeq database^1^. Specificity was verified using the Primer-BLAST program^2^. Primers were synthesized by EUROFINS genomics (Ebersberg, Germany). In addition to electrophoresis in 1% agarose gel, PCR products were also verified by sanger sequencing (EUROFINS). In case of rat *c-Fos*, sequences were adapted from published data (Barros et al., 2015), while in case of the human *c-FOS*, the primer pair was designed *de novo* using Primer-Blast. The *Icer* primers amplificated two of three possible transcript variants of CREM (2, 5). The optimal annealing temperature, the amplification efficiency and *R*^2^ of the standard curve were determined for each primer pair (Table 1) and calculated as follow. PCR efficiency of each primer pair was evaluated by performing a dilution series of the target assay and standard curve analysis of the *Cq* data points using MS excel. The slope of the standard curve (*E* = [10^(–1/^*^*m*^*^)^] − 1 with *E* = amplification efficiency, *m* = slope) was used to calculate the amplification efficiency, which ranged from 0.88 to 1.

**TABLE 1:** Genes and corresponding oligonucleotide primer sequences for qPCR.

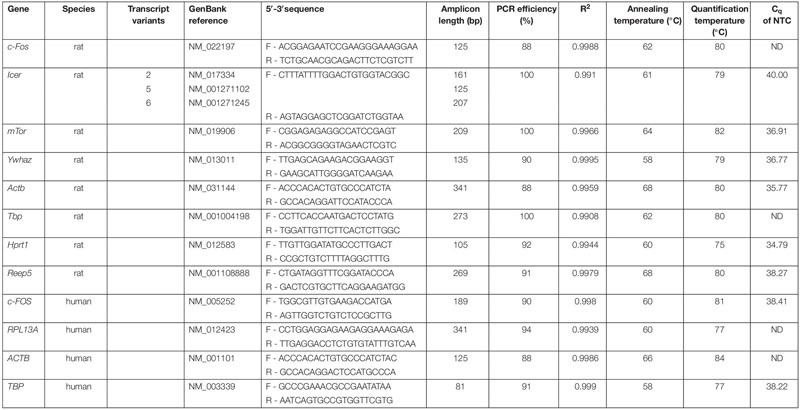

*Gene name, species of the gene and corresponding primer pair, transcript variants, GenBank reference and sequence of the primers are listed. The table shows also the length of the amplicon for each primer pair. Amplification efficiency and *R*^2^ from the standard curve were calculated as described in materials and methods section. Annealing temperature and quantification temperature specific for each primer pair are also listed. F, forward primer; R, reverse primer; bp, base pair; NTC, no template control; ND, not detectable (>40 *Cq*).*

### NormFinder Analysis

The NormFinder algorithm was used to assess expression stability in a set of candidate genes and to identify the most suitable reference gene for RT-qPCR analysis (Andersen et al., 2004). For rat tissue, five candidate reference genes were selected based on literature: beta-actin (*Actb*), tyrosine 3-monooxygenase/tryptophan 5-monooxygenase activation protein (*Ywhaz*), hypoxanthine phosphoribosyltransferase 1 (*Hprt1*), receptor expression-enhancing protein 5 (*Reep5*), and TATA box binding protein (*Tbp*) (Sadangi et al., 2017). NormFinder analysis was separately performed for the three anatomical ROI (H, EC, and TC). To consider all experimental conditions, gene expression was determined in two independent samples of the following nine groups: basal; control 1, 2, 3, and 4 h; 4-AP 1, 2, 3, and 4 h. For human tissue, three candidate genes were selected: *ACTB*, *TBP* and ribosomal protein L13A (*RPL13A*) (Vandesompele et al., 2002; Rydbirk et al., 2016). NormFinder analysis was performed considering all four experimental conditions and examined in two independent samples of the following groups: basal, control, onset site and rest. NormFinder MS Excel application was used to calculate both the intra- and intergroup variation expressed as standard deviation. Candidate genes with a standard deviation > 0.25 were excluded from the subsequent determination of gene stability value, the remaining candidate genes were ranked according to their gene stability value with increasing values implying less stability.

### Quantitative PCR

Quantitative PCR was conducted using the LightCycler 480 II (Roche Diagnostics International AG, Rotkreuz, Switzerland). The reaction assay contained 10 μl LightCycler 480 SYBR Green I Master (Roche Diagnostics International AG, Rotkreuz, Switzerland), 1 μl of forward and reverse primers (0.5 μM each), 1 μl of template cDNA and was diluted by 8 μl H_2_O to a final volume of 20 μl. In the case of primer pairs of the human genes, the quantities of forward and reverse primers were lowered to 0.6 μl (0.3 μM each) for *c-FOS* and 0.4 μl (0.2 μM each) for *ACTB* and *RPL13A*, in order to improve their amplification efficiency. All assays included a minus RT-control to verify previous DNase digestion as well as a negative control without cDNA (NTC), to reveal any non-specific amplification, and were replicated twice. Each PCR reaction was pre-incubated at 95°C for 10 min followed by 45 amplification cycles with the following sequence: melting at 95°C for 5 s, annealing at a primer specific temperature for 10 s, elongation at 72°C for 15 s and quantification at a primer specific temperature for 1 s. After the amplification, melt curve analysis was run with the following sequence: denaturation at 95°C for 30 s, annealing at 70°C for 30 s, followed by the acquisition increasing the temperature to 95°C at a transition rate of 0.11°C/s in continuous mode. Relative quantification of mRNA levels was done using the efficiency-adjusted “delta-delta Ct method” (Pfaffl, 2001). According to this approach, we calculated fold induction of our selected genes relative to basal gene expression:

$$R\,a\,t\,i\,o\,\left(\frac{G\,O\,I}{R\,G}\right)=\frac{E_{G\,O\,I}^{△\,C\,q_{G\,O\,I}\,\left(m\,e\,a\,n\,o\,f\,b\,a\,s\,a\,l\,C\,q-x\right)}}{E_{R\,G}^{△\,C\,q_{R\,G}\,\left(m\,e\,a\,n\,o\,f\,b\,a\,s\,a\,l\,C\,q-y\right)}}$$

with GOI = gene of interest, RG = reference gene, *E* = amplification efficiency, *Cq* = quantification cycle, *x* = individual *Cq* of gene of interest, *y* = individual *Cq* of reference gene.

To determine the actual effect of 4-AP induced epileptiform activity on gene expression, the ratio of 4-AP to control was calculated.

### Cell Death Assay

To evaluate the spatiotemporal profile of slice viability, we performed fluorescent staining with propidium iodide (PI), an indicator of cell death (Buskila et al., 2014). 41 brain slices were taken from three rats and alternatingly assigned to the following groups: basal, control 1 h, control 4 h, 4-AP 1 h and 4-AP 4 h. At the end of each experiment, slices were exposed to PI (1 μg/ml) in carbogenated aCSF for 1 h. In 4-AP treated slices, 4-AP (100 μM) was also added to the staining solution. Following PI staining, slices were fixed by paraformaldehyde (4%) in phosphate buffered saline (0.2 M) for 30 min and subsequently incubated in DAPI (2 μg/ml) for 10 min. Images were acquired using an inverted confocal microscope (LSM 700, Zeiss, Oberkochen, Germany) at 20× magnification. DAPI signals were obtained at 470 nm by laser excitation at 405 nm, while PI signals were measured at 617 nm upon excitation at 555 nm. *Z* plane image stacks were acquired at intervals of 2.47 μm beginning at the surface up to 40–50 mm depth. Images were visualized using ZEN software and processed using ImageJ 1.51m9. DAPI and PI/DAPI positive cells were counted in the somata containing areas of the hippocampal formation (st. granulosum of dentate gyrus, st. pyramidale of CA3, CA1 and subiculum) and two cortical depths (layer I–III and layer IV–VI of the EC and TC, respectively) in a visual field sized 500 × 300 μm. Slice viability was assessed as the ratio between PI positive and DAPI positive (dead/total) cells for each investigated region.

### Data Analysis and Statistics

Raw image data were analyzed with ImageJ 1.51m9, MATLAB and MS Excel. Statistical analysis and processing of graphs were performed using GraphPad Prism 5 or 7 (GraphPad Software, La Jolla, CA, United States). All data were first tested for normality distribution with D’Agostino and Pearson omnibus normality test. If the evaluation of normality was not possible due to too small sample size, Kruskal–Wallis non-parametric test was used. To assess statistical significance between groups, continuous variables were examined using ordinary one-way ANOVA with multiple comparisons (normally distributed data) or Kruskal–Wallis test (non-normally distributed data or small sample size) and *post-hoc* Tukey’s or Dunnett’s multiple comparison of individual groups. Values of *p* < 0.05 were considered statistically significant. All data analyzed by parametric tests are expressed as mean ± standard deviation while data analyzed by non-parametric tests are shown as median ± interquartile range. OmniGraffle 7 (The Omni Group, Seattle, WA, United States) was used as graphical software to process images.

## Results

### Properties of 4-AP Induced SLEs in Acute Rat Brain Slices

**FIGURE 2:**
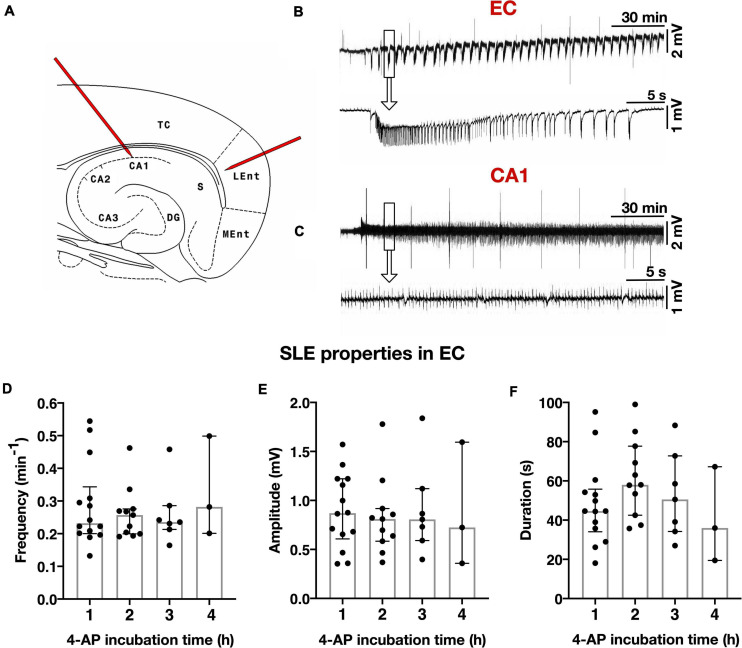
Electrophysiological recordings in rat slices. **(A)** Schematic drawing of a combined hippocampal-entorhinal cortex slice with localization of two extracellular recording electrodes in layer IV/V of the lateral EC and stratum pyramidale of the hippocampal CA1 region, respectively. TC = temporal cortex, LEnt = lateral entorhinal cortex, MEnt = medial entorhinal cortex, S = subiculum, CA1–3 = hippocampal cornu ammonis, DG = dentate gyrus. **(B)** Exemplary trace of local field potentials in the EC during a 4 h experiment with stable occuring seizure-like events (SLEs). Single SLE marked in rectangle with enlarged presentation below. Monomorphic configuration of SLE with an initial sharp transient superimposed by a tonic and then clonic-like phase. **(C)** Representative recording of simultaneous interictal spikes in hippocampal CA1 during the same experimental period, enlarged depiction in the lower trace. **(D–F)** SLE properties in the EC remain stable as demonstrated by similar frequency **(D)**, amplitude **(E)**, and duration **(F)** for each treatment duration in a total number of *n* = 20 slices (five slices for each 4-AP treatment duration of 1, 2, 3, or 4 h). Scatter plots represent individual recordings for 4-AP treatment duration of 1–4 h, superimposed boxes show median ± interquartile range.

**FIGURE 3:**
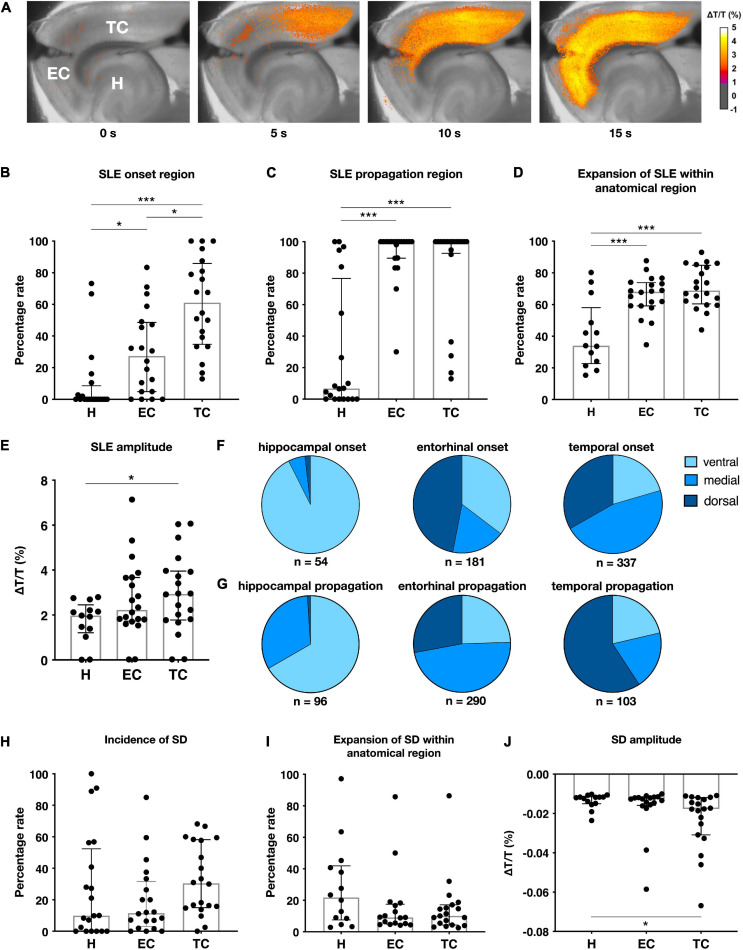
Intrinsic optical imaging (IOS) in rat slices. **(A)** Color-coded SLE amplitude (Δ*T*/*T*) over time during an exemplary SLE generated in the temporal cortex and propagating to the entorhinal cortex without further invading the hippocampal formation. Characteristics of SLEs **(B–E)** and spreading depolarizations (SDs) **(H–J)** measured by IOS in *n* = 20 slices (five slices for each 4-AP treatment duration of 1, 2, 3, or 4 h), scatter plots represent means of individually recorded slices, superimposed boxes show median ± interquartile range (*p < 0.05, ***p < 0.001). **(F–G)** SLE origin is influenced by the anatomical slice origin (dorsoventral axis) separated in ventral (−7.6 to −6.8 mm ventrally from bregma), medial (−6.4 to −5.6 mm) and dorsal (−5.2 to −4.4 mm) regions.

Epileptiform activity due to 4-AP treatment was induced in 100 rat slices (five slices per animal). Local field potentials were recorded in 20 selected slices and yielded 572 SLEs. In the lateral EC, SLE activity started on average 18.0 min (±7.5 min) after 4-AP onset and maintained a stable frequency throughout the entire 4 h incubation period. SLEs displayed the following electrophysiological properties: incidence median 0.24 ± 0.09/min in EC and 0.22 ± 0.05/min in CA1; amplitude median 0.81 ± 0.52 mV in EC and 0.62 ± 0.29 mV in CA1; duration median 50.54 ± 28.39 s in EC and 51.88 ± 40.31 s in CA1 (Figure 2). SLE activity never occurred in slices incubated in standard aCSF. In five ventral slices with respect to the dorsoventral axis, the 4-AP induced activity differed such that no separate SLE but rather a persistent epileptiform activity was observed in the course of the experiment (Supplementary Figure 1). These slices were excluded from further electrophysiological analysis. Simultaneous to electrophysiological recordings, the spatiotemporal evolution of SLEs was monitored by IOS within the entire slice (Figure 3A). Each SLE was assessed for onset and propagation region, respectively. SLE onset in the TC was observed in 61.1% of SLEs followed by 27.4% in the EC and 0.0% in the H (median numbers). In addition to single regions, 57 SLEs (10.0%) in 13 slices had a multiregional onset in anatomical regions distant from each other. The onset region was dependent on the slice origin with respect to the dorsoventral axis such that hippocampal onset almost exclusively occurred in ventral slices while TC and EC onset was more frequent in medial and dorsal slices. Regarding propagation, 49 SLEs (8.6%) in nine slices stayed limited to the onset region while the remaining (>90%) SLEs propagated at least to one neighboring region. The vast majority of SLEs propagated within neocortical regions and invaded hippocampal structures only to a minor degree (6.7%), and, similar to onset, mainly in ventral slices. Propagation to the TC and EC was frequently observed in dorsal and medial slices, respectively. SLE extent assessed by comparing the affected area with the total size of a given region revealed the largest expansion of epileptiform activity in the TC (68.8%) followed by the EC (68.1%). In line with the low rate of onset and propagation, expansion of SLEs within the hippocampus was low (34.0%) and involved in most of the cases the subiculum as previously reported (Heuzeroth et al., 2019). All SLEs were associated with an increase in light transmittance with the maximum in the TC (ΔT/T = 2.94%) followed by the EC (ΔT/T = 2.23%) and the H (ΔT/T = 1.98%). Summary of optical results is given in Figures 3B–G. Overall, the TC stood out such that it presented as the most frequent onset region, displayed the largest intraregional expansion and showed the maximal increase of light transmittance.

### Spreading Depolarizations

Increasing excitability not only decreases the threshold for SLEs but also for spreading depolarizations (SDs) that reflect a propagation of neuronal silencing due to loss of ion homeostasis and a depolarization block (Dreier, 2011). Electrophysiologically, SDs are distinguished from SLEs by their massive amplitude, duration > 1 min and subsequent block of interictal activity. 16.5% of SLEs were associated with simultaneous SDs. No preferred region of SD occurrence could be detected (median numbers, Figure 3H). The relative SD expansion within a given anatomical region was smaller than SLE expansion and showed no significant interregional difference (Figure 3I). In IOS recordings, SDs led to a marked decrease of light transmittance which was pronounced in the TC (ΔT/T = −1.77%) followed by EC (ΔT/T = −1.27%) and H (ΔT/T = −1.19%) (Figure 3J).

### Cell Death Assay

**FIGURE 4:**
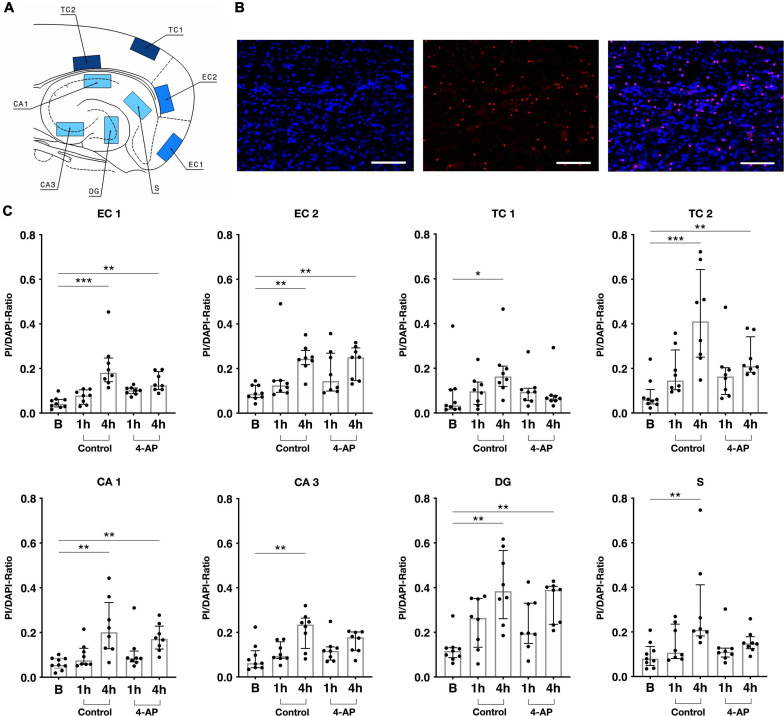
Cell death assay for rat slices. **(A)** Schematic depiction of a rat slice with eight visual fields (500 × 300 μm) in which cells were counted: two layers of each entorhinal (EC1 and EC2) and temporal cortex (TC1 and TC2) as well as subiculum (S), CA1, C3 and dentate gyrus (DG). **(B)** Exemplary images acquired by an inverted confocal microscope (20× magnification, merged *Z* plane image stack), left: DAPI staining (2 μg/ml), in center: PI staining (1 μg/ml), right: merged. Scale bar 100 μm. **(C)** Slice viability measured as ratio of PI (dead) and DAPI (total) positive cells in *n* = 41 slices (9 × basal, 8 × control 1 h, 8 × control 4 h, 8 × 4-AP 1 h, 8 × 4-AP 4 h). Scatter plots represent PI/DAPI-Ratio of individually stained slices, superimposed boxes show median ± interquartile range (*p < 0.05, **p < 0.01, ***p < 0.001).

Neuronal activity in acute brain slices can be recorded up to 8 h after slice preparation. Within this time frame, occurrence of cell death with impact on gene expression and neuronal activity is likely (Buskila et al., 2014) and needs to be considered. To assess the impact of cell death on our results, we performed combined PI and DAPI staining in acute brain slices stored for different time periods. In all investigated brain regions, cell death increased time-dependently under 4-AP treatment as well as control conditions (Figure 4): In basal slices not subjected to storage, the rate of cell death ranged between 3.4% (±8.8%) in the superficial TC layers and 11.6% (±4.5%) in the dentate gyrus. Upon incubation for 4 h (5.5 h when including recovery), the extent of cell death markedly increased. In the hippocampus it varied between 14.8% (±5.4%) in subiculum and 39.0% (±17.1%) in dentate gyrus. In neocortical regions, incubation increased the rate of cell death in deep as well as superficial layers (layer I-III: 15.9% in EC; 9.7% in TC, layer IV-VI: 24.9% in EC; 25.5% in TC). No differences were observed when comparing 4-AP treatment and control, suggesting that 4-AP and the associated epileptiform activity do not increase processes leading to cell death. 4-AP treatment even tended to reduce the cell death rate within the 4 h intervention period when compared to time-matched controls.

### Reference Gene Identification

To identify a reference gene not dependent on neuronal activity or storage duration, we compared *Actb*, *Ywhaz*, *Hprt1*, *Reep5*, and *Tbp*. *Cq* variability did not differ across investigated regions (H: 19.1–32.8; EC: 19.5–29.8; TC: 19.6–31.5) (Supplementary Figure 2A). In all investigated regions, *Actb* was the most abundantly expressed gene and *Tbp* showed the lowest gene expression (Supplementary Figure 2B). In order to obtain a more robust stability ranking, the NormFinder algorithm was applied to calculate the gene stability value M based on standard deviation expressed intra- and intergroup variation (Andersen et al., 2004). Reference genes with standard deviation > 0.25 (*Reep5* in H and *Tbp* in TC) were excluded. Among the remaining candidates, *Ywhaz* turned out to be the most stably expressed gene across all groups (stability value M: H 0.069; EC 0.09; TC 0.071) (Supplementary Figure 2C) and was therefore used as the reference gene for qPCR in the following experiments.

### Gene Expression Analysis

**FIGURE 5:**
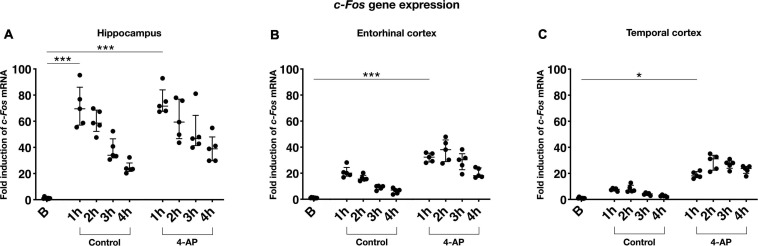
Activity and time dependent *c-Fos* gene expression in rat slices. Induction of *c-Fos* mRNA relative to basal gene expression in **(A)** Hippocampus, **(B)** Entorhinal cortex, and **(C)** Temporal cortex, respectively. All investigated conditions are represented: control slices (control), 4-AP treated slices (4-AP) with experimental duration of 1, 2, 3 or 4 h. For relative quantification in RT-qPCR, *Ywhaz* was used as reference gene. Scatter plots show means of individual rats (*n* = 20) since tissue was pooled before RT-qPCR, superimposed lines represent median ± interquartile range (*p < 0.05, ***p < 0.001).

**FIGURE 6:**
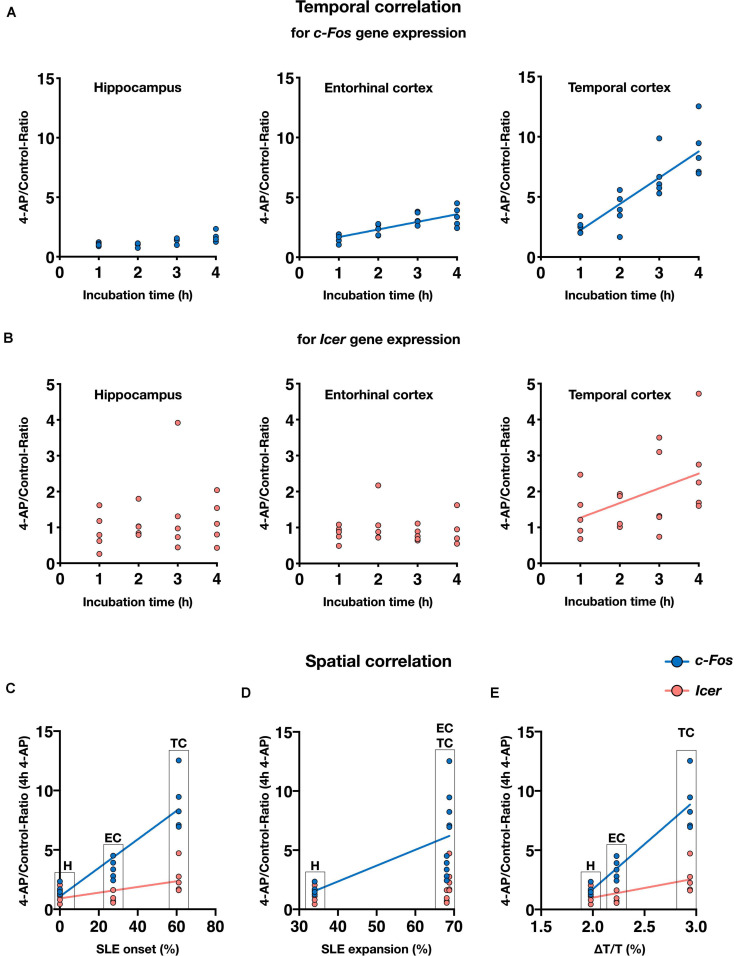
Spatiotemporal correlation of epileptiform activity and gene expression in rat slices. Summary of results from rat slices demonstrating spatial and temporal dependence of *c-Fos* and *Icer* expression. **(A,B)** Scatter plots show means of mRNA expression ratios of 4-AP vs. control from individual rats (*n* = 20) and different incubation intervals for hippocampus (H), entorhinal cortex (EC), and temporal cortex (TC), respectively. Regression lines show significant temporal correlation between 4-AP exposure and *c-Fos* **(A)** or *Icer* expression **(B)**. **(C–E)** Spatial dependence of gene expression following 4-AP treatment duration of 4 h (*n* = 5) shown by spatial parameters such as rate of SLE onset **(C)**, SLE expansion within anatomical region **(D)** as well as SLE amplitude **(E)**. Note that data in C-E are presented as median and do not sum up to 100%.

Investigation of activity-dependent gene expression in rat brain slices was performed for the genes *c-Fos*, *Icer*, and *mTor* referenced to *Ywhaz* expression as stated above. In basal slices immediately processed after the slice procedure, relative *c-Fos* expression varied between regions with the lowest expression in the H and the highest expression in the TC (median *c-Fos/Ywhaz*: *H* = 0.0033, EC = 0.0080, TC = 0.0134). Basal *c-Fos* mRNA levels were positively correlated with the duration of the brain slice preparation (Supplementary Figure 3). In comparison to basal values, incubation in aCSF markedly increased *c-Fos* gene expression. This increase was strongest after 1 h of incubation (corresponding to 2.5 h when including recovery) and most distinctive in the H (median fold induction of 69.5 relative to basal) followed by the EC (median fold increase of 20.1) and the TC (median fold increase of 7.8). Compared to 1 h, incubation for longer intervals (2–4 h or 3.5–5.5 h including recovery) resulted in weaker *c-Fos* expression which was negatively correlated with incubation time (Figure 5). In 4-AP treated slices, a similar time course of *c-Fos* expression with the strongest increase after 1 h of incubation and subsequent decrease was observed. Strikingly, when comparing *c-Fos* expression in 4-AP treated slices to time-matched controls, *c-Fos* expression was consistently higher in the intervention group (Figure 5). Correlation of the duration of intervention and *c-Fos* expression revealed a time-dependent increase of *c-Fos* mRNA that largely differed across examined brain regions (Figure 6A). In the hippocampus, 4-AP treatment increased *c-Fos* mRNA levels only marginally with a (non-significant) slope of 0.19 while significant slopes of 0.64 (*p* < 0.05) and 2.18 (*p* < 0.01) were observed in the EC and TC, respectively. The spatiotemporal pattern of *Icer* expression was similar to *c-Fos* but showed a weaker increase. In all investigated regions of basal slices, *Icer* mRNA levels were low when compared to *c-Fos* mRNA (median *Icer/Ywhaz H* = 0.0002, EC = 0.0003, TC = 0.0003). Reflecting the effect of slice preparation and storage, *Icer* gene expression was also induced in control slices. In contrast to *c-Fos*, the strongest increase of *Icer* mRNA levels was observed in control slices incubated in aCSF for 3 h (median fold induction relative to basal: H 15.1; EC 15.0; TC 6.3) (Supplementary Figures 4A–C). Comparing *Icer* expression between 4-AP and control, slices revealed a region and time-dependent increase of *Icer* mRNA in 4-AP treated slices (Figure 6B): while in the H and the EC, 4-AP treatment had only a marginal effect, it clearly induced an increase in *Icer* expression in the TC with a significant slope of 0.41 (*p*-value 0.0271). In basal slices, *mTor* mRNA did not largely differ between the investigated regions (median *mTor/Ywhaz H* = 0.0137, EC = 0.0151, TC = 0.0145). Interestingly, in all investigated regions, a weak, time-dependent decrease of *mTor* expression in both control and 4-AP treated slices was observed. In the EC and TC, the decrease of *mTor* mRNA levels got significant in 4-AP treated slices after 4 h (Supplementary Figures 4D–F). Compared to time-matched control slices, 4-AP induced epileptiform activity did not show any effect on *mTor* gene expression.

So far, our data demonstrated that gene expression of all examined genes depends on the duration of storage and in case of *c-Fos* and *Icer* additionally to the chosen condition (control/intervention). Gene expression was also associated with the pattern of epileptiform activity: in case of *c-Fos*, the 4-AP induced increase of gene expression and the rate of SLE onset were positively correlated with highest values in the TC and lowest values in the H (Figure 6C). This positive correlation was also observed between *c-Fos* expression and the intraregional SLE expansion and the maximum increase of IOS intensity (Figures 6D–E). Similar effects could be confirmed for *Icer* (Figures 6C–E), albeit to a weaker extent. Naturally, no such correlation was observed for *mTor* that did not show changes in gene expression.

### Gene Expression Analysis in Human Tissue

**FIGURE 7:**
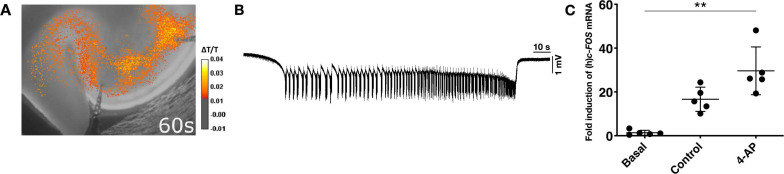
Activity-dependent *c-FOS* expression in human slices. **(A)** Example of a typical IOS image of a SLE in a human cortex slice at 60 s after start. **(B)** Example of an electrophysiological trace showing a SLE recorded in human cortical layers II/III upon application of aCSF containing 8 mM potassium and 100 μM 4-AP. **(C)** Scatter plots showing *c-FOS* expression fold changes relative to basal condition. All data are normalized to the reference gene *RPL13A* and expressed as median ± interquartile range. *c-FOS* expression in slices displaying SLE activity is higher when compared to basal condition (**p < 0.01, *n* = 5).

In human tissue, the NormFinder algorithm revealed *RPL13A* as the most stable gene (stability values M: *RPL13A* 0.068; *ACTB* 0.07; *TBP* 0.115) (Supplementary Figure 5) and therefore employed as reference gene for the following qPCR experiments. To identify the region of seizure onset in human slices, we likewise performed electrophysiological recordings combined with optical imaging (Figure 7A). During 4 h of application, SLEs (Figure 7B) were recorded for a minimum of 40 min to identify the seizure onset site. In a significant proportion of investigated slices, the onset site varied in the course of the experiment. In these slices, the area with the highest number of SLE onsets was assigned as the onset region and further processed. qPCR revealed that *c-FOS* expression increased both during 4 h storage in standard aCSF (control) and epileptogenic aCSF (intervention). Similar to rat slices, the relative increase of *c-FOS* in the intervention group was higher compared to control and basal conditions (Kruskal–Wallis test, *p*-value 0.0031; Dunnet’s *post hoc* test, Basal vs. Onset + Rest *p*-value < 0.01). Values from onset site and the remaining slice were pooled as no difference in *c-FOS* expression could be detected between these regions. As depicted in Figure 7C, in slices subjected to seizure-like activity, *c-FOS* expression was increased 26.1-fold relative to basal conditions. In contrast, control slices incubated with standard aCSF showed a median fold-increase of *c-FOS* expression of 15.7.

## Discussion

In the present proof-of-principle study, we investigated the association between neuronal activity and gene expression in acute brain slice preparations, mainly from rodents. For the activity-dependent genes *c-Fos* and *Icer*, we found a positive spatiotemporal correlation between gene expression and epileptiform activity while no correlation was found for *mTor.* Increases induced by neuronal activity could be differentiated from increases inherent to slice preparation, temperature variations and incubation time. We were able to partly reproduce the results on *c-Fos* mRNA increase observed in rat slices in human brain slice preparations from epilepsy surgery.

The highest correlation between gene expression and epileptiform activity was observed in case of *c-Fos*, its expression is well established as a marker of neuronal activity (Willoughby et al., 1995; Mihály et al., 2001; Szyndler et al., 2009; Barros et al., 2015). As an immediate early gene within the family of inducible transcription factors, *c-Fos* is involved in regulating cellular responses including differentiation, plasticity and cell degeneration (Chiu et al., 1988; Pennypacker et al., 1994). Proconvulsant stimuli result in an increase of *c-Fos* mRNA as well as c-FOS protein in rat brains *in vivo* (Barros et al., 2015; Yang et al., 2019). A regional and persistent induction of *c-FOS* gene expression was also found in human epileptic neocortex regardless of the underlying pathology (Rakhade et al., 2005). So far, only little data exists about *c-Fos* induction in brain slices (Massamiri et al., 1994), and this work suffered from technical limitations as changes in *c-Fos* expression were assessed semiquantitatively using southern blots without accounting for differences between subregions and impact of exposure time.

With a weaker extent than *c-Fos*, SLE activity was also associated with increased *Icer* mRNA. *Icer* is a member of cAMP-dependent transcription factors (*Crem* = cAMP response element modulator). Electroconvulsive seizures *in vivo* increased *Crem* and *Icer* mRNA in rat brains with a maximum at 1 h after seizures and with a most prominent increase in the dentate gyrus and deep layers of cerebral cortex (Fitzgerald et al., 1996). In a different model using pilocarpine-induced status epilepticus in rats, *Icer* mRNA assessed in the dentate gyrus peaked at 6 h and declined to control levels after 1 week (Lund et al., 2008). Interestingly, phosphorylation of cAMP response element-binding protein (CREP) that is assumed to positively affect *ICER* gene expression was regionally increased in seizure onset zone of human neocortex measured following epilepsy surgery (Rakhade et al., 2005), suggesting that involvement of the *CREM/ICER* pathways is likely in the pathophysiology of human epilepsy. Several studies support the idea that activation of the *ICER* pathway upon seizures represents an adaptive mechanism aiming to reduce excess neuronal activity. *Crem/Icer* null mutant mice suffered from more frequent seizures following status epilepticus than their control littermates (Porter et al., 2008), and overexpression of *Icer* negatively regulated neuronal plasticity (Kojima et al., 2008). By revealing a positive correlation of *Icer* expression and epileptiform activity, we were able to reproduce these results *in vitro* specifically in the TC.

Finally, we investigated gene expression of *mTOR*, a member of a complex regulatory pathway modulating proliferation and neuronal activity. In patients with tuberous sclerosis complex suffering from severe and treatment-resistant epilepsy, the *mTOR* pathway is hyperactivated due to mutations in TSC1 and TSC2 genes (Overwater et al., 2019). In these patients, treatment with *mTOR* inhibitors such as everolimus reduced seizure frequency in up to 40% of individuals (French et al., 2016). Apart from tuberous sclerosis complex, the involvement of the *mTor* pathway in structural epilepsy was shown in several animal models (Huang et al., 2010; Zhang and Wong, 2012; Wang et al., 2018), including chronic models induced by kainate and pilocarpine. Importantly, the acute phase of *mTor* activation seems to be directly driven by epileptiform activity. Pentylenetetrazole (PTZ)-induced seizures caused a transient *mTor* activation in rat hippocampus and neocortex starting by 1 h and returning to baseline by 16 h after seizure onset (Zhang and Wong, 2012). Activation of the *mTor* pathway is believed to occur by protein phosphorylation rather than gene activation (Zeng et al., 2009; Zhang and Wong, 2012). In our study, we did not find evidence for additional activation of *mTor* gene expression by epileptiform activity. Rather conversely, *mTor* expression seems to be negatively affected by *in vitro* storage as demonstrated by decreased *mTor* mRNA levels in the entorhinal and temporal cortex.

The results from our cell death assay based on PI/DAPI staining imply that the 4-AP *in vitro* model represents a suitable tool to examine gene expression *in vitro*. Although cell death expectedly increased with increasing incubation intervals, we did not observe spatial or temporal differences between control and intervention groups. Interestingly, 4-AP treatment for 4 h tended to decrease the cell death rate when compared to time-matched controls. Previous studies demonstrated that preconditioning with 4-AP for 48 h protects rat cellular granule neuron culture against excitotoxicity by a number of stressors (glutamate, NMDA and 3-nitropropionic acid) (Smith et al., 2009). The results are consistent with an earlier investigation of 4-AP induced protection of hippocampal and cortical neuron culture (Hardingham et al., 2002; Tauskela et al., 2008). Overall, exposure to 4-AP or other proconvulsant agents such as the GABA_*A*_-receptor antagonist bicuculline, seems to be involved in neuroprotection (Hardingham et al., 2002; Tauskela et al., 2008), although the exact mechanisms remain unclear.

In our approach, we aimed to systematically overcome previous technical limitations by using various internal controls, applying quantitative means to measure gene expression and comparing different regions within a slice as well as different exposure times to the applied proconvulsant. As an acute *in vitro* model of seizures, we used 4-AP as in this model epileptiform activity remains stable over hours. We could clearly detect spatial differences demonstrated by the highest increase of *c-Fos* expression in the TC, the most frequent onset region for SLEs, while in the hippocampus, only a marginal increase in *c-Fos* mRNA was observed. This correlation applied also to the intraregional SLE expansion and activity-dependent increase of light transmittance.

Our work poses several limitations. According to previous reports, hippocampal slice preparation causes a remarkable induction of *c-Fos* mRNA within 6 h when compared to intact hippocampus (Taubenfeld et al., 2002). We confirmed these results with a hippocampal *c-Fos* mRNA increase up to 70-fold while increase in the entorhinal and temporal cortex was much lower. We assume that structural differences between the investigated regions including strong reciprocal connectivity in the hippocampus renders the latter more sensitive to the transient but massive neuronal activation during slice preparation. This high sensitivity with massive *c-Fos* increase in basal hippocampal slices presents a limiting factor as gene expression is not unlimited. It seems rpossible that strong initial hippocampal *c-Fos* increase made a further increase upon SLE induction less likely. This aspect together with the low incidence of SLEs in the hippocampal formation might explain the weak effects of SLE activity on hippocampal gene expression. The artificial, *in vitro* setting presents a general limitation that cannot be easily overcome. Gene expression observed *in vitro* might largely differ from the natural *in vivo* situation. Therefore, future observations from human samples investigated *in vitro* need to be interpreted with caution.

In human slices, *c-FOS* expression correlated with epileptiform activity, but we did not find increased expression in the onset zone, most likely due a high variability of the SLE onset and consequently probable imprecise definition. This onset variability as compared to rat tissue is likely due to the overall variability of human tissue obtained from different patients with consequent impact on the activity *in vitro* (Andersson et al., 2016; Wickham et al., 2018; Kraus et al., 2019) and also due to differences in tissue or different slicing angles (Kraus et al., 2020). All these variables not only influence neuronal survival and network excitability, but also could have affected gene expression. Finally, the infrequent availability and low number of human samples allowed only for a limited analysis and also precluded detailed comparison with rat tissue.

The discussed limitations need to be considered in future studies. In case of human tissue, however, using an *ex vivo* approach remains without alternatives. Overall, our results indicate that irrespective of limitations mostly imposed by *ex vivo* conditions, *in vitro* models in general represent a suitable tool for the investigation of gene expression by epileptiform activity. As an outlook, transcriptome-wide approaches including spatial transcriptomics as well as investigation of single cell profiles might reveal novel candidates involved in the human pathophysiology of epilepsy and possibly other CNS pathologies.

## Data Availability Statement

All datasets generated for this study are included in the manuscript and/or the Supplementary Files.

## Ethics Statement

The studies involving human participants were reviewed and approved by Charité-Universitätsmedizin Berlin (EA2/111/14). The patients/participants provided their written informed consent to participate in this study. The animal study was reviewed and approved by the Institutional Animal Welfare Officer and the responsible local authority (Landesamt für Gesundheit und Soziales Berlin, T0336/12).

## Author Contributions

SS, PF, and MH contributed to the conception and design of the study. SS performed and analyzed all experiments in rat brain slices, including electrophysiological and optical recordings, cell death assay, qPCR, and NormFinder analysis. MH selected the patients for operation. JO operated the patients. LM performed and analyzed all measurements in the human tissue. MD-W and PF contributed to the data analysis and statistics. SS, LM, and PF wrote the manuscript, which all authors edited and finalized. All authors contributed to the article and approved the submitted version.

## Conflict of Interest

The authors declare that the research was conducted in the absence of any commercial or financial relationships that could be construed as a potential conflict of interest.

## Footnotes

1 www.ncbi.nlm.nih.gov

2 www.ncbi.nlm.nih.gov/tools/primer-blast/index.cgi

## Supplementary Material

Supplementary Figure 1 Persistent epileptiform activity in ventral rat slices. Exemplary electrophysiological recording of 4-AP induced persistent epileptiform activity in the EC observed ventral rat slices (−7.6 to −6.8 mm from bregma) in contrast to separate SLEs in medial and dorsal slices.

Supplementary Figure 2 Reference gene identification for rat slices. **(A)** Mean quantification value (*Cq*) and standard deviation of five reference genes determined in two independent samples for each condition (basal; control 1, 2, 3, and 4 h; 4-AP 1, 2, 3, and 4 h; *n* = 18). Values obtained from the three rat brain regions processed: hippocampus, entorhinal cortex and temporal cortex. **(B)** *Cq* of five candidate reference genes in all conditions across the three regions investigated (hippocampus, *H*, entorhinal cortex, EC, and temporal cortex, TC). The box chart indicates the first and the third interquartile range. The vertical line across the box indicates the median, while the lower and upper dashes show the minimum and maximum values. **(C)** Expression stability values (*M*) of the five candidate reference genes evaluated by NormFinder. A lower stability value indicates a more stable expression. *Reep5* in hippocampus and *Tbp* in temporal cortex showed a standard deviation > 0.25, so they were excluded from the determination of the stability value.

Supplementary Figure 3 Gene expression of *c-Fos* in basal rat slices. Duration of brain slice procedure (from decapitation to cell lysis in TRIzol) positively correlated with basal *c-Fos* mRNA levels. Relative to the reference gene Ywhaz, *c-Fos* expression in basal slices was highest in temporal cortex (TC) followed by entorhinal cortex (EC) and hippocampus (H) in decreasing order.

Supplementary Figure 4 Gene expression of *Icer* and *mTor* in rat slices. Fold induction of *Icer* **(A–C)** and *mTor* **(D–F)** relative to basal gene expression in hippocampus, entorhinal, and temporal cortex, respectively. All investigated conditions are represented: control slices (control) and 4-AP treated slices (4-AP) with experimental duration of 1, 2, 3, or 4 h. For relative quantification in RT-qPCR, *Ywhaz* was used as reference gene. Scatter plots show means of individual rats (*n* = 20) since tissue was pooled before RT-qPCR, superimposed graphs represent median ± interquartile range (^∗^*p* < 0.05, ^∗∗^*p* < 0.01, ^∗∗∗^*p* < 0.001).

Supplementary Figure 5 Reference gene identification for human slices. **(A)** Mean quantification value (*Cq*) and standard deviation of three reference genes determined in two independent samples for each condition considered for human samples (basal, control, onset site, rest of slice; *n* = 8). **(B)** *Cq* of three candidate reference genes in all conditions. The box chart indicates the first and the third interquartile range. The vertical line across the box indicates the median, while the lower and upper dashes show the minimum and maximum values. **(C)** Expression stability values (*M*) of the three candidate reference genes evaluated by NormFinder. A lower stability value indicates a more stable expression.
