# Supplementary figures and images for: Spatiotemporal Correlation of Epileptiform Activity and Gene Expression in vitro
Source: Front Mol Neurosci. 2021 Mar 30;14:643763. doi: 10.3389/fnmol.2021.643763 (PMC8042243; doi:10.3389/fnmol.2021.643763)

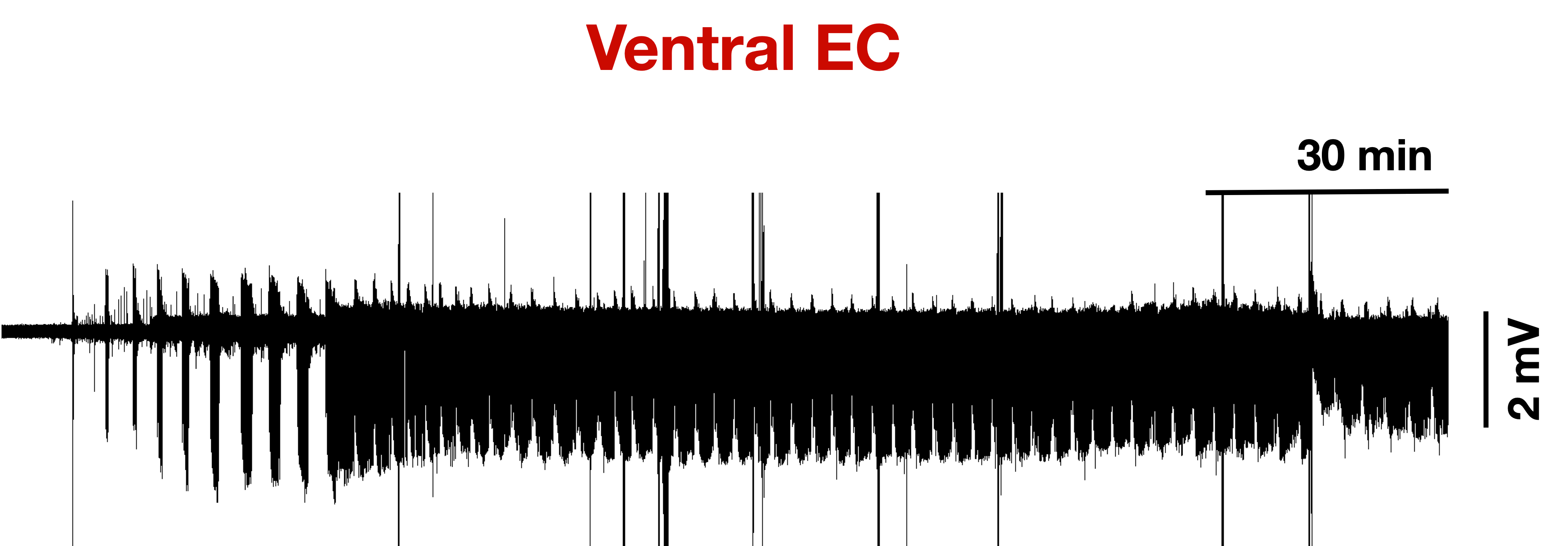

Supplement: Supplementary Figure 1 — Persistent epileptiform activity in ventral rat slices. Exemplary electrophysiological recording of 4-AP induced persistent epileptiform activity in the EC observed ventral rat slices (−7.6 to −6.8 mm from bregma) in contrast to separate SLEs in medial and dorsal slices. [file Image_1.TIFF]

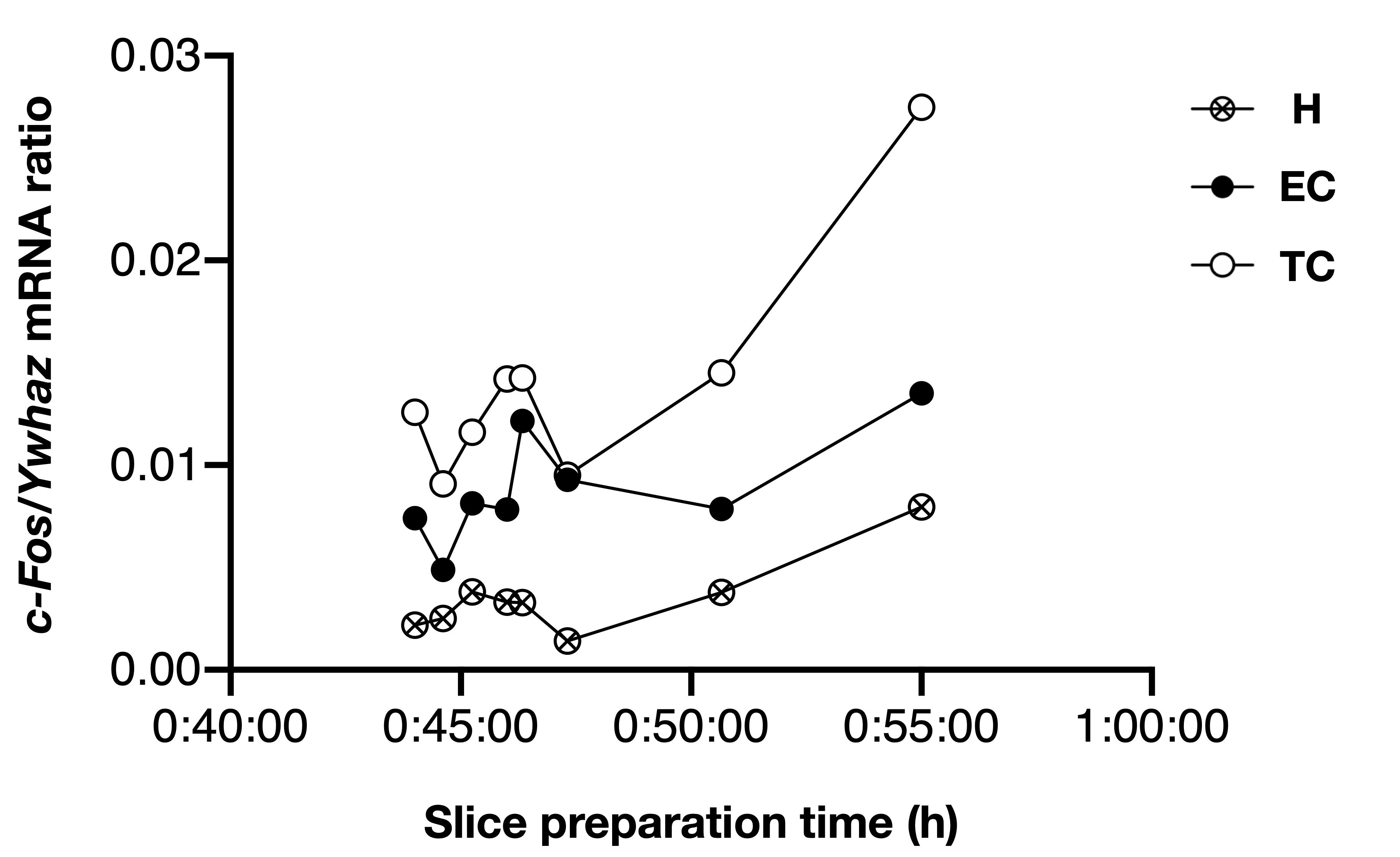

Supplement: Supplementary Figure 3 — Gene expression of c-Fos in basal rat slices. Duration of brain slice procedure (from decapitation to cell lysis in TRIzol) positively correlated with basal c-Fos mRNA levels. Relative to the reference gene Ywhaz, c-Fos expression in basal slices was highest in temporal cortex (TC) followed by entorhinal cortex (EC) and hippocampus (H) in decreasing order. [file Image_3.TIFF]

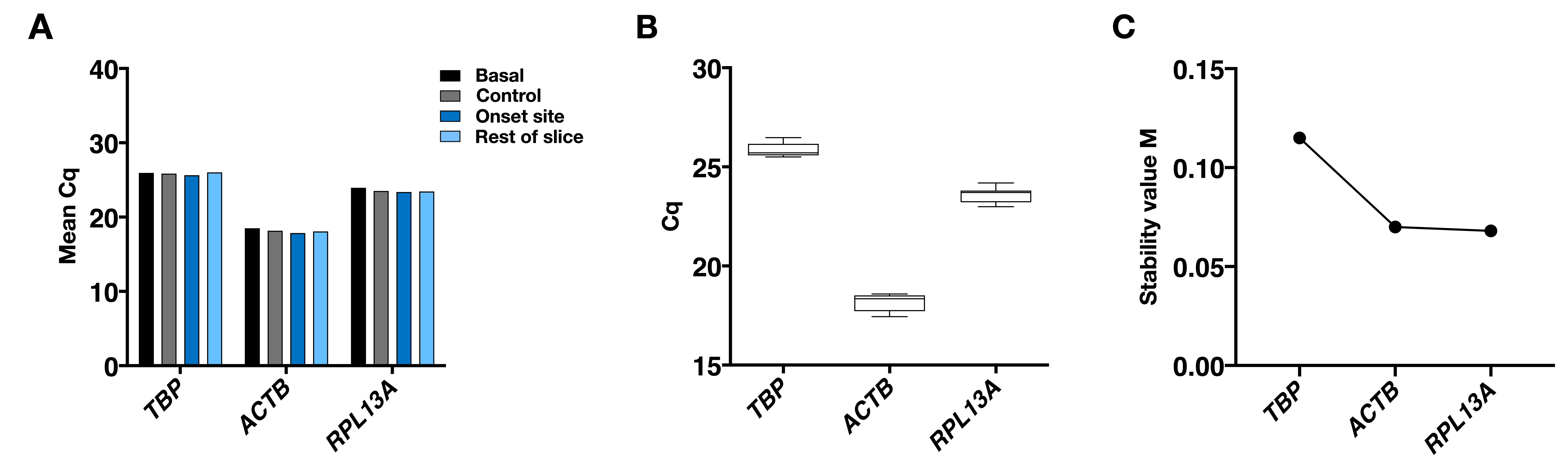

Supplement: Supplementary Figure 5 — Reference gene identification for human slices. (A) Mean quantification value (Cq) and standard deviation of three reference genes determined in two independent samples for each condition considered for human samples (basal, control, onset site, rest of slice; n = 8). (B) Cq of three candidate reference genes in all conditions. The box chart indicates the first and the third interquartile range. The vertical line across the box indicates the median, while the lower and upper dashes show the minimum and maximum values. (C) Expression stability values (M) of the three candidate reference genes evaluated by NormFinder. A lower stability value indicates a more stable expression. [file Image_5.TIFF]
